# Dengue Infection and Its Relationship with Evans Syndrome: A Pediatric Case

**DOI:** 10.1155/2021/8635585

**Published:** 2021-08-12

**Authors:** Ivan Jose Ardila Gomez, Pilar Pérez López, Mónika Rocío Hernández Carreño, Juan Camilo Barrios Torres

**Affiliations:** ^1^Pediatric Critical Care, Uros Clinic-Neiva University Hospital, Professor at the Universidad Surcolombiana, Neiva, Huila, Colombia; ^2^Pediatric Rheumatologist, Neiva University Hospital-Uros Clinic-Medilaser Clinic, Professor at the Universidad Surcolombiana, Neiva, Huila, Colombia; ^3^Pediatrician and Epidemiologist Uros Clinic Medilaser Clinic, Neiva, Huila, Colombia; ^4^General Physician Specialist in Health Quality Management and Audit, Hospital Medical Coordinator of Uros Clinic, Neiva, Huila, Colombia

## Abstract

Dengue is a single-stranded RNA virus belonging to the Flaviviridae family. It is an endemic virus in tropical countries. In Colombia, 4 serotypes are present, and the disease is a burden for public health, social programs, and the economic sectors. The main vector is *Aedes aegypti*, and most infections are asymptomatic or minimally symptomatic. The hemorrhagic appearances of severe dengue are due to plasma leakage as a result of increased vascular permeability, severe thrombopenia, and hemoconcentration. In 2020, 78,979 cases of dengue were reported in Colombia. 38,836 (49.2%) of them were warning-free signs, 39,246 (49.7%) with warning signs, and 897 (1.1%) of severe dengue. As it is well-known, viral diseases are immune system activators, triggering off a loss of tolerance in it. Dengue is not an exception, and it is able to explain different autoimmune phenomena including macrophage activation. Mechanisms have been described by which an exacerbated response of the disease is triggered through the increase of infected cells, formation of immune complexes, and complement pathway activation, which lead to a cross-reaction of viral antigens with epithelial cells with platelets with subsequent endothelial dysfunction and bleeds. The first description of Evans syndrome was made in 1951 by Robert Evans. This syndrome is characterized by the combination of autoimmune hemolytic anemia, immune thrombocytopenia, and, less common/usual, immune neutropenia. This disease's etiology is unknown, and the dysregulation of the immune system is among its possibilities. Here, we present the case of an unusual hematological and immunological complication of a patient who developed Evans syndrome during severe dengue, taking into account the concomitantly limited literature available for these two diseases, the need for a broader diagnostic approach, multidisciplinary intervention, and a more complex therapeutic approach.

## 1. Introduction

Dengue is a single-stranded RNA virus belonging to the Flaviviridae family. It is an endemic virus in tropical countries. In Colombia, 4 serotypes are present (DENV1, DENV2, DENV3, and DENV4) and the disease is a burden for public health, social programs, and the economic sector. It is transmitted in two ways: the vertical one (from mother to child) and the vector through the bite of infected mosquitoes [1], and it has also been reported during transfusion of blood components and organ transplant. The main vector is *Aedes aegypti*, although transmission through the *Aedes albopictus* mosquito is also described in the literature, which has a lower transmission capacity [2, 3], and most infections are asymptomatic or minimally symptomatic. The hemorrhagic appearances of severe dengue are due to plasma leakage as a result of increased vascular permeability, severe thrombopenia, and hemoconcentration.

In 2020, 78,979 cases of dengue were reported in Colombia. 38,836 (49.2%) of them reported warning-free signs, 39,246 (49.7%) with warning signs, and 897 (1.1%) of severe dengue. Dengue incidence in Colombia on a regional basis is 295.2 cases per 100,000 inhabitants at risk. For Huila, incidence rates higher than 500 cases per 100,000 inhabitants are estimated [4].

Dengue is a disease that has a wide spectrum of signs affecting different organs and systems; sometimes, special therapies are required to maintain the proper functioning of the different affected organs. Among the main cardiovascular complications of dengue, it is the dengue shock, which is considered as the clearest way of plasma leakage and the main cause of the potential fatal outcome of the disease. This is an acute state of cardiovascular dysfunction leading to an inability to carry enough amounts of oxygen and nutrients to meet metabolic needs [5]. As it is well-known, viral diseases are immune system activators, triggering off a loss of tolerance in it. Dengue is not an exception, and it is able to explain different autoimmune phenomena including macrophage activation. Mechanisms have been described by which an exacerbated response of the disease is triggered through the increase of infected cells, formation of immune complexes, and complement pathway activation, which lead to a cross-reaction of viral antigens with epithelial cells and platelets with subsequent endothelial dysfunction and bleeds [6].

The first description of Evans syndrome was made in 1951 by Evans et al. [7, 8]. This syndrome is characterized by the combination of autoimmune hemolytic anemia, immune thrombocytopenia, and, less common/usual, immune neutropenia [9–14]. This disease's etiology is unknown, and the dysregulation of the immune system is among its possibilities [11, 13, 15].

Here, we present the case of an unusual hematological and immunological complication of a patient who developed Evans syndrome during severe dengue, taking into account the concomitantly limited literature available for these two diseases, the need for a broader diagnostic approach, multidisciplinary intervention, and a more complex therapeutic approach.

## 2. Clinical Case

**Figure 1:**
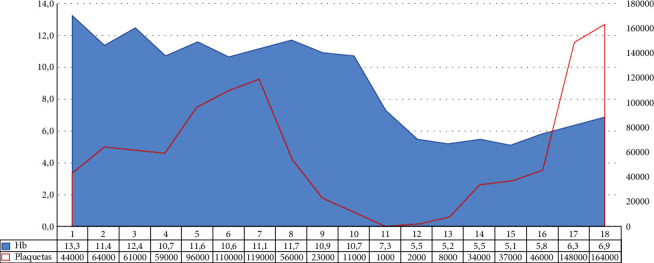
Platelet and hemoglobin behavior of the patient with severe dengue and Evans syndrome.^*∗*^Hemoglobin levels are shown in area form, and their values appear on the left axis ^*∗*^^*∗*^Platelet levels are shown as a line, and their values are shown on the right-hand axis.

A 6-year-old male patient (weight: 19 kg, SD: −0.70; height 113 cm, SD: −0.56; BMI: 14.9 SD: −0.41) with no significant medical history, coming from the first level of care with clinical manifestation of 5-day evolution, consistent nonquantified fever, asthenia, adynamia, myalgia, arthralgia, emetic episodes, abdominal pain, and headache. The patient was hospitalized with a probable dengue diagnosis with warning signs. He shows a torpid clinical course, with epistaxis, progressive thrombopenia, dengue hepatitis, oligoanuria, 20% right pleural effusion, fever persistence, and hepatosplenomegaly. The patient was taken to the pediatric ICU due to his proven severe dengue diagnosis. In follow-up tests, positive IgM serology for dengue, hematological affection was documented due to bicytopenia (anemia and thrombopenia) without evidence of external or hidden bleeding (Figure 1) requiring platelet transfusion on two occasions; it was decided to transfuse because the patient had already overcome the dengue critical phase, and this decrease was progressive until high-bleeding risk values from vital organ are associated with severe hemodynamic affection, secondary to Evans syndrome and not to a hematological manifestation of dengue.

Given the atypical course of the disease, an interdisciplinary approach was used on the part of the pediatric subspecialties of infectious diseases, hemato-oncology, and rheumatology. Studies were prolonged including autoimmunity profile, bone marrow aspiration, and biopsy (findings: nonnecrotizing epithelioid granuloma), polycultures (negative blood cultures 1 and 2, negative urine culture, and negative myeloid culture), negative infectious profile (IgM and IgG serologies for negative *Toxoplasma gondii*; negative cytomegalovirus; negative Epstein–Barr; negative hepatitis A, B, and C studies; nonreactive HIV ELISA; and nonreactive VDRL), and biomarkers (positive Coombs test). The patient was considered to have Evans syndrome, and treatment with immunoglobulin was suggested, 2 g/kg/total dose, with a partial response and subsequently a steroid cycle with prednisolone, a dose of 1 mg/kg/day initially with programmed titration until suspended. Outpatient follow-up was carried out for 6 months after discharge, and the patient was discharged due to pediatric hemato-oncology and rheumatology. Clinical improvement was documented with supervision of the described condition without relapse on cytopenia and nonadministration of the oral steroid.

## 3. Discussion

The first description of Evans syndrome was made in 1951 by Robert Evans, who studied 24 patients with ages between 3 and 78 years [7, 8]. This disease consists of the combination of autoimmune hemolytic anemia, immune thrombopenia and less usual, immune neutropenia (between 15 and 55%) and can be classified as primary or secondary [9–14]. Its etiology is unknown and among the possible causes it is the dysregulation of the immune system which leads to the production of IL-10 and interferon gamma, leading to activation of Autoreactive T Lymphocytes and production of autoantibodies by B lymphocytes [11, 13, 15].

In the literature review, reports of dengue hemolytic anemia cases are considered to be of low prevalence. These reports are about adult patients. In the description by Aye et al., it is reported a male patient who develops hemolytic anemia during the dengue recovery phase. This finding could be related to the severity of the disease and this etiological agent should be taken into account as a cause of autoimmune hemolytic anemia, so much that it is compared with Mycoplasma pneumoniae [12]. The case report by Kulkarni and Sharma describes a 52-year-old patient with Dengue and warning signs. Hemolytic anemia is subsequently documented with a positive Coombs test. In general, patients with dengue have anemia due to viral medullary aplasia and documented bleedings; having hemolytic anemia is considered an atypical manifestation of the disease [16].

Although the diagnostic criteria are clearly described, underreporting may take place in relation to the number of cases diagnosed and published since, in some situations, the direct Coomb's test may be negative. One reason is because in some commercial antiglobulin reagents, the IgG sensitization is below the detection threshold. Another cause is that if the optimal conditions are not found in the analytical phase, for example, if the preparatory wash is not carried out at 4°C or with a low ionic strength, the elimination of the low affinity IgG can be generated. As a third cause, sensitization of red blood cells with just one IgA or with low molecular weight (monomeric) IgM, not accompanied by complement fixation, may result in a negative test since a large variety of commercial antiglobulin reagents have only anti-IgG and anti-C3 [17].

Nowadays, treatment is based on immunomodulatory therapy schemes depending on the clinical conditions of the patient and comorbidities. The first line of treatment includes oral corticosteroids at a dose of 1-2 mg/kg/day and IV immunoglobulin at a dose of 2 g/kg/total dose; both options were used in this case for the management of cytopenia secondary to Evans syndrome. In severe cases, the use of steroids is recommended at initial doses of 4–6 mg/kg/day during the first 72 hours [18]. Although transfusions are not recommended due to the risk of exacerbation, they are only prescribed in cases of severe hemodynamic affection and risk of vital organ affection, as it is evidenced in our case [13]. The second line of management is the immunosuppressive drugs: azathioprine, cyclosporine, mycophenolate, ofetil, vincristine, danazol, sirolimus, cyclophosphamide, and rituximab or surgical management with splenectomy, especially in patients with frequent relapses or in those who receive high doses of corticosteroid aimed at reducing doses and minimize their side effect [11, 14, 15]. In severe and refractory cases, hematopoietic stem cell transplant is considered [9, 13].

In the multicenter study published by the French group led by Aladjidi, conducted at the Bordeaux University Hospital, 156 patients were analyzed in 26 clinics during the period from 1981 to 2014. Given the characteristics of this cohort and the disease severity, azathioprine and rituximab were used as a second line of treatment [8].

About 50% of Evans syndrome cases are associated with autoimmune diseases such as systemic lupus erythematous, lymphoproliferative diseases, and common variable immunodeficiency, with a higher prevalence in the pediatric population [8, 9]. Some immune deficiencies can occur with autoimmune manifestations without active infections; cases with immunological alterations have been documented in controls after the first-line management (steroids or immunoglobulin), proving the diagnosis of mainly humoral immunodeficiency [10, 14].

When faced with an endemic tropical disease in many regions with a wide spectrum of clinical and paraclinical manifestations and with a wide range of severe complications, we think it is important to identify unusual clinical patterns that require timely interventions to avoid definitive secondary injuries or fatal outcome. Until the date of completion of this paper, no case was found in the most important international, regional, and local databases describing the association between both diseases in pediatric patients; therefore, we consider this to be the first case reporting an association between dengue disease and Evans syndrome in a child.

## Data Availability

The data can be accessed by searching the references included in the current case report.
